# Prenatal Hypoxia–Ischemia Induces Abnormalities in CA3 Microstructure, Potassium Chloride Co-Transporter 2 Expression and Inhibitory Tone

**DOI:** 10.3389/fncel.2015.00347

**Published:** 2015-09-03

**Authors:** Lauren L. Jantzie, Paulina M. Getsy, Jesse L. Denson, Daniel J. Firl, Jessie R. Maxwell, Danny A. Rogers, Christopher G. Wilson, Shenandoah Robinson

**Affiliations:** ^1^Department of Pediatrics, University of New Mexico, Albuquerque, NM, USA; ^2^Department of Neurosciences, University of New Mexico, Albuquerque, NM, USA; ^3^Department of Neurosurgery, Boston Children’s Hospital, Harvard Medical School, Boston, MA, USA; ^4^Department of Neurology, Boston Children’s Hospital, Harvard Medical School, Boston, MA, USA; ^5^Department of Pediatrics, Case Western Reserve University School of Medicine, Cleveland, OH, USA; ^6^Department of Pediatrics, Center for Perinatal Biology, Loma Linda University, Loma Linda, CA, USA; ^7^F.M. Kirby Center for Neurobiology, Boston Children’s Hospital, Harvard Medical School, Boston, MA, USA

**Keywords:** epilepsy, hippocampus, KCC2, microstructure, prematurity, seizure

## Abstract

Infants who suffer perinatal brain injury, including those with encephalopathy of prematurity, are prone to chronic neurological deficits, including epilepsy, cognitive impairment, and behavioral problems, such as anxiety, inattention, and poor social interaction. These deficits, especially in combination, pose the greatest hindrance to these children becoming independent adults. Cerebral function depends on adequate development of essential inhibitory neural circuits and the appropriate amount of excitation and inhibition at specific stages of maturation. Early neuronal synaptic responses to γ-amino butyric acid (GABA) are initially excitatory. During the early postnatal period, GABA_A_R responses switch to inhibitory with the upregulation of potassium-chloride co-transporter KCC2. With extrusion of chloride by KCC2, the Cl^−^ reversal potential shifts and GABA and glycine responses become inhibitory. We hypothesized that prenatal hypoxic–ischemic brain injury chronically impairs the developmental upregulation of KCC2 that is essential for cerebral circuit formation. Following late gestation hypoxia–ischemia (HI), diffusion tensor imaging in juvenile rats shows poor microstructural integrity in the hippocampal CA3 subfield, with reduced fractional anisotropy and elevated radial diffusivity. The loss of microstructure correlates with early reduced KCC2 expression on NeuN-positive pyramidal neurons, and decreased monomeric and oligomeric KCC2 protein expression in the CA3 subfield. Together with decreased inhibitory post-synaptic currents during a critical window of development, we document for the first time that prenatal transient systemic HI in rats impairs hippocampal CA3 inhibitory tone. Failure of timely development of inhibitory tone likely contributes to a lower seizure threshold and impaired cognitive function in children who suffer perinatal brain injury.

## Introduction

Although improved obstetrical and neonatal intensive care practices have led to increased survival, infants born very preterm are prone to disorders of cerebral development, including impaired cognition and behavior, epilepsy, and cerebral palsy (Marin-Padilla, 2000; Robinson et al., 2005; Volpe, 2009). Similarly, infants who suffer brain injury from hypoxia–ischemia (HI) during critical developmental periods of cerebral circuit formation are also at increased risk for seizures, neuropsychiatric conditions, and cognitive disorders (Martinez-Biarge et al., 2010). Altered intrinsic neuronal network activity, including formation of aberrant or excess local connections, and significant disruption of the excitatory–inhibitory developmental program, are among the theories proposed to explain the predisposition to hyperexcitability following preterm birth and perinatal HI (Marin-Padilla, 2000; Robinson, 2005).

Improper levels of cerebral excitatory and inhibitory tone likely contribute to spasticity, epilepsy, and other neurological deficits associated with prematurity. Inhibition and excitation are inextricably interwoven during development and in the mature CNS (Isaacson and Scanziani, 2011), and KCC2 appears to synchronize this balance (Li et al., 2007). During postnatal development KCC2 regulates maturation of inhibitory neurotransmission and tone (Rivera et al., 1999; Hubner et al., 2001; Payne et al., 2003; Dzhala et al., 2005; Kanold and Shatz, 2006; Daw et al., 2007; Farrant and Kaila, 2007). KCC2 extrudes chloride, and maintains the Cl^−^ gradient responsible for hyperpolarization observed following γ-amino butyric acid (GABA)_A_ and glycine receptor activation (Rivera et al., 1999; Kaila et al., 2014a). Immature neurons typically have higher intracellular Cl^−^ compared to mature neurons, as a result of low KCC2 membrane expression (Rivera et al., 1999; Dzhala et al., 2005). Increased KCC2 expression promotes membrane hyperpolarization and enhanced inhibitory responses from GABA_A_ receptor activation, thus supporting the generation of inhibitory post-synaptic currents (IPSCs) (Farrant and Kaila, 2007), and formation of inhibitory cerebral circuits (Daw et al., 2007). Indeed, inhibitory tone alters action potential propagation. Spontaneous IPSCs suggest activation of post-synaptic GABA_A_ receptors following action potential-dependent vesicular transmitter release (Alvarez-Dolado et al., 2006). Notably, increases in spontaneous IPSC frequency often reflect increased inhibitory tone (Alvarez-Dolado et al., 2006). KCC2 and GABA_A_R maturation are linked in models of CNS injury and repair (Papp et al., 2008; Jantzie et al., 2015; Tian et al., 2015).

Numerous factors regulate the rapid increase in cerebral KCC2 expression during the perinatal period, including subplate neurons (Kanold and Shatz, 2006; Jantzie et al., 2015). Subplate neuronal loss is a central component of CNS injury from preterm birth (Volpe, 1996; Kinney et al., 2012; Pogledic et al., 2014). Indeed, post-mortem specimens from preterm infants with white matter injury have reduced KCC2 cerebral expression (Robinson et al., 2010), consistent with the hypothesis that inadequate KCC2 expression during the critical period of cerebral circuit development contributes in part to impaired inhibitory tone in preterm infants. Prenatal transient systemic hypoxia–ischemia (TSHI) on embryonic day 18 (E18) in Sprague-Dawley rats models CNS injury associated with extreme preterm birth (Robinson et al., 2005). Following this injury, adult rats have a lower seizure threshold induced by the GABAergic antagonist pentylenetetrazol (Mazur et al., 2010). Given that prenatal TSHI mimics multiple components of CNS injury from very preterm birth (Robinson et al., 2005; Mazur et al., 2010; Jantzie et al., 2015), we hypothesized that TSHI would impair developmental KCC2 upregulation in CA3, reduce IPSCs during a critical period of circuit formation, and lead to chronic abnormalities in CA3 microstructure. Specifically, we predicted that prenatal TSHI would lower inhibitory tone during the first two postnatal weeks and that this functional impairment would correlate with reduced KCC2 expression and abnormalities on diffusion tensor imaging (DTI).

## Materials and Methods

All procedures were performed in accordance with the NIH Guide for the Care and Use of Laboratory Animals and with the approval of the Animal Care and Use Committees at Case Western Reserve University, Boston Children’s Hospital and the University of New Mexico.

### Prenatal transient systemic hypoxia–ischemia

The neurodevelopmental pattern of expression of neurotransmitters, receptors, and co-transporters is staggered in rodents and humans. In general, full-term in the Sprague-Dawley rat is approximately equivalent to 30 weeks gestation in humans, while postnatal day 7 (P7) in rats is similar to full-term in humans. The period of rapid increase in KCC2 expression in the early postnatal rat corresponds to the third trimester in humans (Robinson et al., 2010; Hyde et al., 2011; Kaila et al., 2014a), a period of vulnerability of cerebral circuit development in preterm infants (Robinson, 2005). Here, an established model of prenatal TSHI injury was used (Robinson et al., 2005; Jantzie et al., 2013, 2014, 2015). Briefly, on embryonic day 18 (E18) Sprague-Dawley rats were anesthetized with isoflurane. A laparotomy was performed, uterine arteries were clamped for 60 min, and the laparotomy was closed. Sham control dams underwent anesthesia and laparotomy for 60 min but uterine arteries were not clamped. All pups were born at term and matured with their respective dams. Both sexes were used in all experiments.

### Diffusion tensor imaging

By measuring tissue integrity at the micron level, *ex vivo* DTI allows quantification of microstructural injury (Aung et al., 2013). Observed patterns of abnormalities vary with type of insult (Sierra et al., 2015), and recovery intervals following injury (Mac Donald et al., 2007). To assess long-term abnormalities in CA3 hippocampal microstructure, rats at P35–40 were deeply anesthetized with sodium pentobarbital and perfused with 4% paraformaldehyde. Brains were then removed and after post-fixation, embedded in 2% agarose containing 3 mM sodium azide for *ex vivo* magnetic resonance imaging (MRI). MRI was performed on a Bruker 4.7-T BioSpec 47/40 Ultra-Shielded Refrigerated nuclear system equipped with a 72 mm I.D. quadrature RF coil and a small-bore (12 cm I.D.) gradient set with a maximum gradient strength of 50 Gauss/cm. MR protocols consisted of echo-planar diffusion tensor imaging (EP-DTI) sequences. Images of 12 contiguous coronal 1 mm slices were obtained with a field-of-view (FOV) of 3.00 cm, a TR of 3000 ms, TE of 40 ms, and *b*-value 2000 mm^2^/s with 30 gradient directions. CA3 was analyzed using Bruker’s *Paravision* 5.1 imaging software. Diffusion-weighted images and fractional anisotropy (FA) maps were generated. Axial (λ1) and Radial [(λ2 + λ3)/2] diffusivity eigenvectors were also measured by observers blinded to the injury status.

### Immunohistochemistry

Double-labeling immunohistochemistry was performed at P11 to assess KCC2 loss on neurons in the CA3 subfield. After perfusion with 4% paraformaldehyde, brains were immersed in 30% sucrose. Coronal 20 μm frozen sections were cut on a cryostat. Sections were thawed, and incubated with block consisting of 10% goat serum and 0.5% Triton in phosphate-buffered saline (PBS) for 1 h. Antibodies were diluted in 2% NGS/0.5% Triton/PBS. Sections were incubated sequentially with anti-KCC2 antibodies (1:500, Millipore, Billerica, MA, USA) overnight at 4°C, PBS rinse, anti-rabbit biotinylated IgG antibodies (Vector Labs, Burlingame, CA, USA), fluorescein-conjugated avidin, mouse anti-NeuN antibodies (1:1000, Millipore, Billerica, MA, USA), and AlexaFluor-568 antibodies (Life Technologies, Grand Island, NY, USA). Sections were mounted with Vectashield (Vector Labs, Burlingame, CA, USA). Images were photographed using a Leica DMi8 confocal microscope by observers blinded to the injury group. Images were obtained at 63×, zoom 0.89, with the following laser settings used for all images: seq 1 – 495@ 6.23, gain = 123.75%; seq 2 – 581@ 13.1, gain = 106.25%.

### Western blot

Western blotting for KCC2 was performed on micro-dissected CA3 at P15 (*n* = 8/group). Previously, we have reported the developmental time course of oligomeric and monomeric KCC2 expression from P7 to young adulthood in Sprague-Dawley rats (Jantzie et al., 2014). Briefly, membrane proteins were isolated using a sucrose-containing homogenization buffer, sonication, and differential centrifugation (Jantzie et al., 2014). Protein amount was determined via Bradford protein assay, after which 15 μg was loaded on to 4–20% Tris HCl gels (BioRad Hercules, CA, USA). Following transfer, membrane s were blocked and incubated in anti-KCC2 (1:500, Millipore) overnight at 4°C. The following day, membranes were washed, incubated in anti-rabbit HRP-conjugated secondary antibodies, and developed in Femto-West ECL. Digital images were captured on GE LAS 4000 image reader and resultant bands quantified with ImageQuant software and standardized to beta-tubulin to confirm equal protein among lanes.

### Electrophysiology

Acute tissue slices containing the hippocampus were prepared from P10 to P11 TSHI or sham pups, as described previously (Calcagnotto et al., 2002). Slices were continuously perfused with oxygenated artificial cerebrospinal fluid (ACSF) consisting of (in millimolar), 124 NaCl, 3 KCl, 1.5 CaCl_2_⋅2H_2_O, 1.0 MgSO_4_⋅7H_2_O, 0.5 NaH_2_PO_4_⋅H_2_O, 25 NaHCO_3_, and 30 d-Glucose, pH 7.4 (295–305 mOsm) at 27°C. Coronal (400 μm thick) slices were cut on a VT1000 vibratome (Vibratome Instruments, St. Louis, MO, USA), while continuously perfusing the tissue with chilled (3–6°C) oxygenated (95% O_2_–5% CO_2_) sodium-containing ACSF slicing medium. Final slices were cut to contain the hippocampus for recording from visualized CA3 pyramidal neurons. Following sectioning, the resulting slices were immediately transferred to a holding chamber where they remained submerged in oxygenated (95% O_2_–5% CO_2_) room temperature recording medium (ACSF). For each experiment, a single slice was transferred from the holding chamber to a polycarbonate recording chamber (26GLP, Warner instruments, Hamden, CT, USA) and held in place with a platinum ring overlain with nylon threads. The slice was continually perfused with ACSF bubbled with carbogen gas at room temperature for up to 6 h. Approximately 30 min before whole-cell recordings were performed on CA3 pyramidal neurons, the slice was left undisturbed to equilibrate with the surrounding recording medium.

#### Intracellular Recording

Whole-cell voltage-clamp (Axoclamp, 700A, Molecular Devices, Sunnyvale, CA, USA) recordings were obtained from CA3 pyramidal neurons. Patch pipettes (Borosilicate glass BF150, Sutter Instruments, Novato, CA, USA), pulled to a 1.5–2 μm tip diameter (Sutter Instruments P-97) to give a resistance of 4–6 MΩ, were used to record from individual neurons in the CA3 region of the hippocampus (sampled at 10 kHz, bandpass filtered at 10–1000 Hz). Intracellular patch pipette solution used to study IPSCs contained (in millimolar): 120 Cs-gluconate, 10 HEPES, 11 EGTA, 11 CsCl, 1 MgCl_2_, 1.25 QX-314, 2 Na_2_ATP, 0.5 Na_2_GTP, and pH 7.25 with KOH. Cells exhibiting large leak currents (>100 pA) were excluded from analysis. Cesium can influence KCC2 cation transport (Payne et al., 2003; Williams and Payne, 2004), however, because sham and TSHI slices were evaluated using the same solution, the impact was the same for all slices. In addition, Cs^+^ has been used in recording solutions in prior studies of co-transporter function (Sipila et al., 2009; Zonouzi et al., 2015). IPSCs were recorded for 10 min at a holding potential of 20 mV to correct for the liquid junction potential (Calcagnotto et al., 2002). IPSC V-clamp recordings were recorded using *Clampfit* software (Molecular Devices), and IPSCs exported and analyzed using time-to-peak and time-to-decay parameters in *Mini Analysis* 5.6.28 software (Synaptosoft, Decatur, GA, USA). Briefly, each spontaneous event was manually selected based on the IPSC waveform and rise time, amplitude, and decay properties. Between 100 and 500 individual IPSC events were recorded and analyzed for each cell. Data are presented as the mean ± SEM. Cumulative probability plots and histograms were constructed using Python (http://python.org) scripts and Excel (Microsoft, Redmond, WA, USA).

### Statistical analyses

Data are presented as mean with SEM. Differences were compared using a two-sample two-tailed Student’s *t*-test assuming unequal variance, with a significance level of *p* < 0.05.

## Results

### Microstructural and diffusion abnormalities in the CA3 subfield following prenatal TSHI

**Figure 1:**
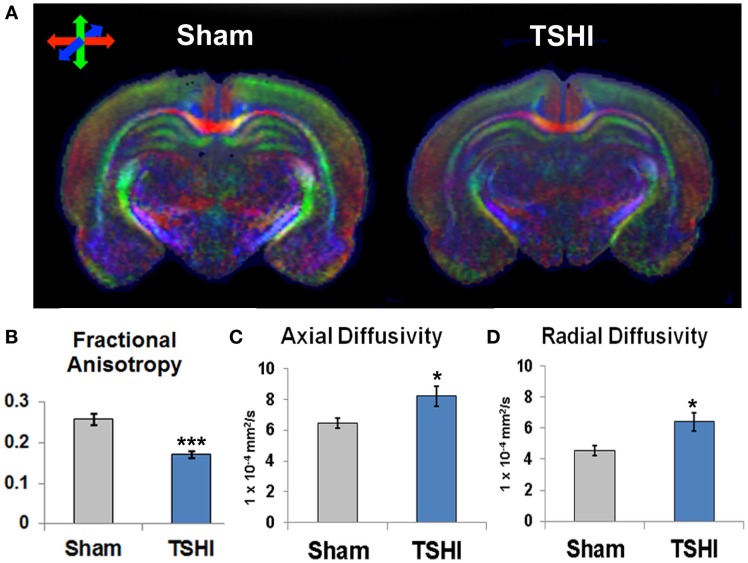
**Diffusion tensor imaging of young mature rats following prenatal TSHI shows microstructural abnormalities**. **(A)** DTI directional diffusion color maps show differences in hippocampal structure in TSHI rats compared to shams. Red color indicates transverse tracts, blue color indicates anterior–posterior tracts, and green color indicates vertical tracts. **(B)** Fractional anisotropy (FA) is reduced in the CA3 subfield of P35–40 TSHI animals compared to shams. **(C)** Axial diffusivity (AD) is increased in CA3 of TSHI brains compared to shams, consistent with axonal injury. **(D)** Radial diffusivity is increased in CA3 of TSHI brains compared to shams in young mature CNS, consistent with impaired myelin integrity. *p < 0.05, ***p ≤ 0.001.

Diffusion tensor imaging of microstructural abnormalities measures structural damage at the micron level, particularly related to the integrity of axons and myelin (Aung et al., 2013). DTI color maps from juvenile brains (P35–40) following prenatal TSHI demonstrate marked loss of directional diffusion in the three primary directions and integrity compared to shams (Figure 1A). Microstructural abnormalities were quantified. Following prenatal injury, juvenile TSHI animals show significantly reduced FA compared to shams (*n* = 7–8/group, *p* < 0.001, Figure 1B), suggesting loss of structural integrity from early injury persists into the mature CNS. In agreement with the loss of structural integrity observed with FA, axial diffusivity and radial diffusivity are both increased in TSHI rats compared to shams, consistent with injury to axons and myelin, respectively (AD: *p* = 0.04 and RD: *p* = 0.02, Figures 1C,D). These data show that prenatal TSHI causes sustained DTI microstructural abnormalities in the CA3 subfield.

### Prenatal TSHI reduces KCC2 expression in the CA3 subfield

**Figure 2:**
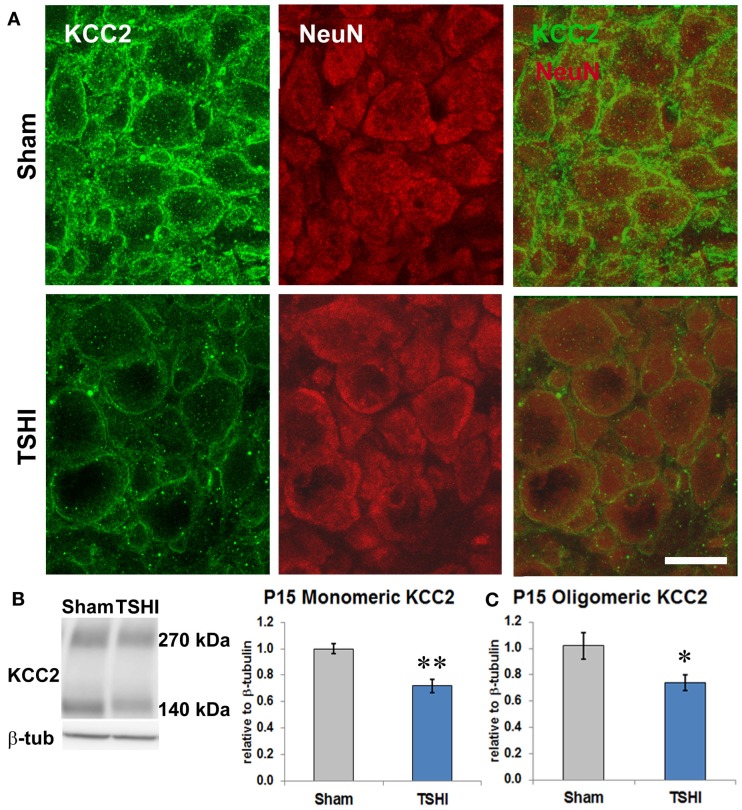
**Prenatal TSHI diminishes KCC2 expression during postnatal development in the CA3**. **(A)** NeuN-KCC2 double-labeling at P11 shows reduced KCC2 expression. Bar = 20 μm. **(B)** Western blot at P15 shows reduced monomeric KCC2 expression (140 kDa) in CA3 from TSHI brains compared to shams. **(C)** Similarly, oligomeric KCC2 (~270 kDa) expression is also reduced at P15 compared to shams (*p < 0.05, **p < 0.01).

KCC2 expression is susceptible to CNS injury (Galeffi et al., 2004; Bonislawski et al., 2007; Papp et al., 2008; Boulenguez et al., 2010; Jaenisch et al., 2010; Ma et al., 2014; Tian et al., 2015), and reduced KCC2 has been reported in CA3 pyramidal neurons resected from humans with epilepsy (Huberfeld et al., 2007), and in peri-tumoral cortex (Pallud et al., 2014; Campbell et al., 2015). To determine if prenatal TSHI altered CA3 KCC2 expression on CA3 neurons in our model, we performed double-labeling immunohistochemistry. Marked reduction of KCC2 expression on NeuN^+^ neurons is present in the CA3 subfield of P11 TSHI rats, compared to shams (Figure 2A). Western blotting was performed on CA3 membrane fractions to quantify KCC2 expression at P15. In the TSHI CA3 subfield, monomeric and oligomeric KCC2 expression is reduced by 29 and 28%, respectively, compared to shams (*n* = 8/group, *p* = 0.002 and *p* = 0.03, respectively, Figures 2B,C). Together, these results demonstrate that prenatal TSHI diminishes expression of KCC2 in the CA3 subfield, consistent with our prior investigations that reported sustained reductions in KCC2 expression resulting from calpain-mediated degradation (Jantzie et al., 2014).

### Prenatal TSHI decreases IPSC frequency

**Figure 3:**
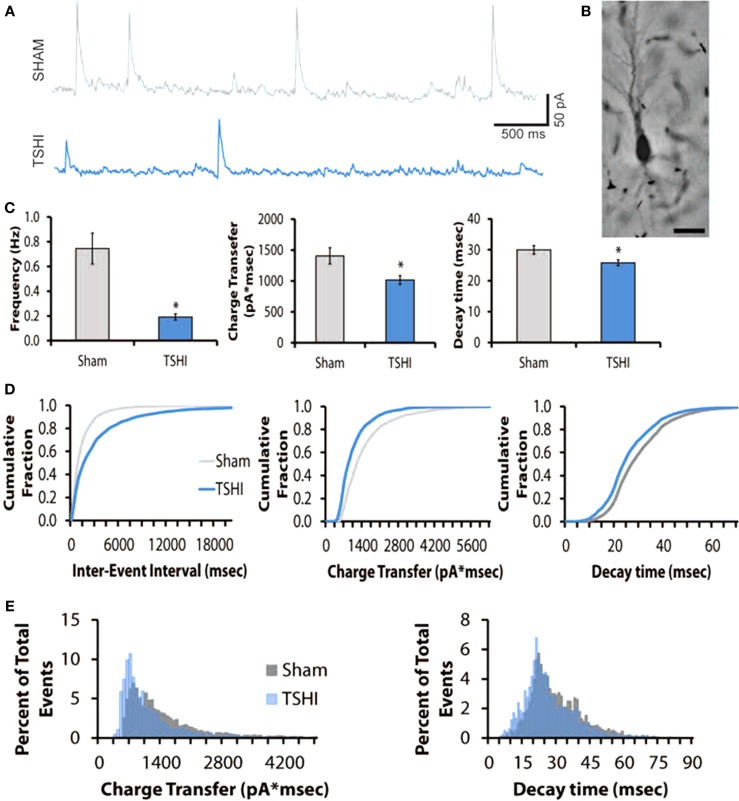
**Transient systemic hypoxia–ischemia (TSHI) decreases inhibitory post-synaptic currents (IPSCs)**. **(A)** Tracing showing decreased frequency of post-synaptic events in TSHI CA3 regions compared to sham; **(B)** biocytin-filled CA3 P10–P11 pyramidal neuron in hippocampal slice. Bar = 50 μm. **(C)** Frequency, charge transfer, and decay of IPSCs are significantly different in TSHI animals compared to shams (two-tailed *t*-test, *p* < 0.05, *p* = 0.006, and *p* = 0.019, respectively). **(D)** Consistent with the mean data, the cumulative distribution plots demonstrate a leftward shift in the population of TSHI IPSCs, and decreased inter-event intervals compared to shams. **(E)** Event distribution histograms confirm the leftward shift in decay and charge transfer following TSHI compared to shams.

Given the contribution of KCC2 to inhibitory tone (Sivakumaran et al., 2015), the reduced KCC2 protein expression in CA3 we observed during a period of rapid and critical circuit development in the first two postnatal weeks (P11–P15), and the lower seizure threshold observed in adult rats following prenatal TSHI (Mazur et al., 2010), we sought to determine if prenatal TSHI affects inhibitory tone. Charge transfer and frequency of IPSCs were measured in hippocampal CA3 pyramidal cells on P10–11 (Figures 3A,B), a period of rapid KCC2 upregulation in the CA3 of Sprague-Dawley rats (Jantzie et al., 2014). Mean IPSC frequency decreased by 60% after TSHI compared to shams (11–12 cells from 6 to 7 rats/group; *p* = 0.006, Figure 3C). Similarly, charge transfer (area) dropped by 28% following TSHI compared to shams (*p* = 0.019, Figures 3C,D), concomitant with a 14% reduction in the time constant of decay after TSHI (*p* < 0.05, Figure 3D). IPSC amplitude also trended down in TSHI animals compared to shams, but did not reach significance (TSHI: 46 pA vs. sham: 56 pA). Concomitant with the mean data, distribution histograms and cumulative distribution plots demonstrate a clear leftward shift in the population of IPSCs from TSHI rats compared to shams (Figures 3D,E). Together, diminished IPSC frequency and charge transfer (Alvarez-Dolado et al., 2006) indicate that CA3 pyramidal neurons were depolarized following prenatal TSHI when compared to sham CA3 neurons, leading to a loss of inhibitory tone and impaired functional refinement of developing circuits.

## Discussion

In the present study, we demonstrate chronic loss of CA3 microstructure, and loss of KCC2 protein expression and IPSCs during a crucial window of postnatal circuit development. Given loss of expression of GABA signaling and KCC2 in premature infants (Robinson et al., 2006, 2010), and the relationship between chloride transporter expression and GABA_A_R maturation (Kanold and Shatz, 2006; Jantzie et al., 2015; Tian et al., 2015), these data emphasize that the multifaceted nature of inhibitory changes in the developing hippocampus following *in utero* injury defined, in part, by alterations in KCC2 expression.

Here, we show for the first time that functional impairment in hippocampal slices, defined by lower inhibitory tone, is present in pyramidal neurons following prenatal injury. Indeed, one facet of inhibitory strength is the chloride electrochemical gradient, which drives the hyperpolarizing action of synaptic inhibition and is determined by intracellular chloride concentration (Yassin et al., 2014). Early in development, inhibitory synapses generate excitatory post-synaptic potentials that stabilize synapse formation, and as neurons mature there is upregulation of KCC2 that drives low intracellular chloride and supports hyperpolarizing IPSCs (Kandler and Gillespie, 2005; Yassin et al., 2014). Notably, the trafficking, cell surface expression, and transport activity of KCC2 are controlled by neuronal activity, with increased KCC2 activity caused by protein oligomerization and changes in phosphorylation (Blaesse et al., 2006; Chamma et al., 2013). Thus, as intracellular chloride declines, the driving force favors influx of chloride ions, which strengthens IPSCs and synaptic inhibition (Ben-Ari et al., 2012; Yassin et al., 2014). In the present study, we demonstrate that prenatal TSHI decreases IPSC frequency, and reduces oligomeric and monomeric KCC2 protein expression during this critical period of maturation. Additionally, we show reduced KCC2 expression on CA3 neurons. Previously, we have shown KCC2 loss following TSHI is caused in part by calpain-mediated degradation rather than overt changes in phosphorylation (Jantzie et al., 2014). Together, these data support the relationship between KCC2 expression and inhibitory activity, and are consistent with the reduced seizure threshold in adult rats following prenatal injury (Mazur et al., 2010). Indeed, calpain-mediated KCC2 loss is consistent with recent reports of reduced KCC2 levels in the chronic period following neonatal seizures (Puskarjov et al., 2015), and excess calpain activity in human epilepsy resections (Feng et al., 2011; Das et al., 2012).

Plasticity in neural circuitry can be due to changes in ion transporters activity, including KCC2 (Kaila et al., 2014b). In addition to controlling the intracellular chloride concentration, KCC2 regulates multiple other components of neurodevelopment and mature CNS function. Indeed, the broad multi-tasking spectrum of KCC2 functions has earned the KCC2 co-transporter the designation of a multi-functional “moon-lighting” protein (Blaesse and Schmidt, 2015). Ion-independent functions include regulating neural tube formation (Horn et al., 2010), synapse formation (Tanis et al., 2009; Blaesse and Schmidt, 2015), interneuron migration (Bortone and Polleux, 2009), neuronal survival (Pellegrino et al., 2011; Winkelmann et al., 2015), and dendritic spine formation (Li et al., 2007; Fiumelli et al., 2013; Llano et al., 2015). Cortical KCC2 expression is also tightly linked with maturation of GABA_A_ receptors (Chudotvorova et al., 2005; Kanold and Shatz, 2006; Jantzie et al., 2015).

Functional regulation of KCC2 expression and activity is complex (Medina et al., 2014). KCC2 activity is modulated by transcription (Uvarov et al., 2006; Markkanen et al., 2008; Ludwig et al., 2011), post-translational modification via phosphorylation (Lee et al., 2010; Kahle et al., 2013), and calpain degradation (Puskarjov et al., 2012; Zhou et al., 2012; Jantzie et al., 2014). Numerous factors contribute to the regulation of KCC2 expression and function. For example, neuroligin-2, a cell adhesion molecule that regulates GABAergic synaptogenesis, also regulates KCC2 expression. Neuroligin-2 expression precedes KCC2 expression during development, and loss of neuroligin-2 delays the GABAergic switch from depolarizing to hyperpolarizing during development (Sun et al., 2013). Interestingly, loss of neuroligin-2 is linked to social dysfunction (van der Kooij et al., 2014) and cognitive impairment (Liang et al., 2015), deficits often found in preterm infants (Anderson, 2014). Likewise, thyroxin is important for BDNF-induced survival of injured neurons (Shulga et al., 2009), and early hypothyroidism prevents upregulation of KCC2 expression from P10 to P15 in Wistar rats (Sawano et al., 2013). Lack of adequate KCC2 expression during the critical period of neurodevelopment may contribute to the profound impact hypothyroidism has on neurodevelopment. Moreover, preterm infants are prone to various types of hypothyroidism (Vigone et al., 2014). Indeed, preventing hypothyroidism may modulate KCC2 expression in preterm infants.

Given the importance of KCC2 to neurodevelopment, particularly cerebral circuit formation, and the multitude of neurological deficits suffered by preterm infants, modulation of KCC2 expression in the critical neonatal period is appealing as a potential therapeutic intervention. Neonatal EPO treatment administered after prenatal TSHI restores the seizure threshold and functional deficits in adults, and promotes neuronal and oligodendroglial survival and maturation (Mazur et al., 2010; Jantzie et al., 2013). Neonatal EPO treatment also limits loss of calpain-mediated KCC2 loss in the CA3 after prenatal injury (Jantzie et al., 2014). In clinical trials of human preterm infants, EPO derivatives improved cognitive outcomes at 2 years (Ohls et al., 2014), and reduced white and gray matter MRI abnormalities at term (Leuchter et al., 2014). A KCC2 enhancer has been described (Gagnon et al., 2013), but its efficacy, and most importantly, safety in the developing brain after injury has yet to be investigated. The premise that we can reverse the consequences of KCC2 loss and its functional deficits is exciting, but the use of such agents relies on the appropriate testing in clinically relevant injury models, and thoughtful interpretation and integration of the findings.

A limitation of this study is that we did not have an adequate sample size to clarify sex differences in inhibitory tone. Developmental upregulation of KCC2 expression varies between male and female Wistar rats (Murguia-Castillo et al., 2013), and neonatal allopregnanolone promotes KCC2 expression in male rats (Modol et al., 2014); however, sex differences in KCC2 have not been studied in Sprague-Dawley rats. In this model, males tended to show a lower seizure threshold than females, although both males and females were significantly lower than shams (Mazur et al., 2010). These findings are consistent with human preterm infants, where being male is a risk factor for a worse neurodevelopmental outcome (Ambalavanan et al., 2012; Kent et al., 2012). Second, regional variation in KCC2 expression and activity likely dictates functional outcomes (Kovacs et al., 2014; Yang et al., 2015). Encephalopathy of prematurity affects the entire developing CNS (Volpe, 2009). Indeed, widespread loss of KCC2 expression was found in post-mortem samples from preterm infants with white matter lesions (Robinson et al., 2010), and in rats following prenatal TSHI (Jantzie et al., 2015). Here, investigation of inhibitory tone and its relationship to KCC2 expression and microstructural abnormalities was confined to only in the CA3 subfield. Despite these limitations, our study demonstrates loss of KCC2 expression and inhibitory tone in CA3 in the injured developing brain and persistent injury with CA3 microstructural abnormalities in the mature CNS. Given the global prevalence and impact of perinatal brain injury, the findings reported here demonstrate sustained alterations in the developing brain following prenatal HI that provide insight into impaired cerebral circuit formation.

## Author Contributions

All authors contributed in the preparation of the manuscript, and approved the final version of the manuscript. LJ produced, analyzed and interpreted data, and wrote the manuscript. PG, DF, JD, JM, and DR produced and analyzed data. CW supervised electrophysiology data collection, analysis, and interpretation. SR conceived the hypothesis, wrote the manuscript, and supervised all portions of data collection, analysis, and interpretation.

## Conflict of Interest Statement

The authors declare that the research was conducted in the absence of any commercial or financial relationships that could be construed as a potential conflict of interest.

## Funding

Supported by NIH-NINDS R01 NS060765 to Shenandoah Robinson and the Centers for Biomedical Research Excellence Pilot Award to Lauren L. Jantzie (Jantzie/CoBRE P30GM 103400/Pi:Liu).
